# Nutrient assimilation from puffer and tilapia aquaculture sludge by marine polychaete *Neanthes acuminata* (Ehlers, 1868): a way forward to solid waste management

**DOI:** 10.1007/s11356-026-37698-9

**Published:** 2026-04-02

**Authors:** Md. Khorshed Alam, Mana Ito, Hiroaki Shiraishi, Akira Ohtani, Tomohiro Akagi, Yoshinobu Yamagiwa, Katsutoshi Ito

**Affiliations:** 1 Environmental Conservation Division, Environment and Fisheries Applied Research Department, Fisheries Technology Institute, Japan, Fisheries Research and Education Agency, 2-17-5 Maruishi, Hatsukaichi, Hiroshima 739-0452 Japan; 2 Technical Research Institute, Okumura Corporation, 387 Ohsuna, Tsukuba, 300-2612 Japan; 3 New Business Development Department, Okumura Corporation, Marunuochi JP Tower 22F, Chiyoda-Ku, Tokyo, 100-7022 Japan; 4 Industrial Technology Center of Wakayama Prefecture, 60 Ogura, Wakayama, 649–6261 Japan

**Keywords:** Aquaculture sludge, Polychaete, *Neanthes acuminata*, Nutrient upcycling, Bioremediation, Stable isotope analysis

## Abstract

Despite its contribution to food security, managing solid waste from land-based aquaculture has been a major problem worldwide. Various bioremediation approaches have long been used to manage solid waste. Hence, a holistic and self-driven system is lacking. As a practical alternative, aquaculture sludge can provide habitats and nutrition, thereby increasing biomass production for deposit-feeding polychaetes. As nutrient uptake abilities and reproductive strategies can vary among species, we examined the survival, growth, and nutrient uptake efficiency of *Neanthes acuminata* from sludge types collected separately from two recirculating aquaculture systems: puffer (i.e., *Takifugu rubripes*) and tilapia (i.e., *Oreochromis niloticus*). A higher survival rate (i.e., 80–90%) was achieved in the sludge treatments compared with the negative control over an eight-week period. The growth rate increased in both sludge treatments across the experimental weeks, suggesting their ability to utilize nutrients in metabolic processes. Carbon and nitrogen uptake were evident, with a higher nitrogen percentage. The enrichment of nitrogen stable isotope ratios within polychaete body tissue in puffer sludge exposure further supports the upcycling rate. The carbon-to-nitrogen ratio of fecal materials of *N. acuminata* after 10-day exposure was significantly higher than the initial sludge, indicating the supply of carbon-enriched materials within the sludge substrate. This study confirms the survival, growth, and nutrient-upcycling efficiency of a newly introduced polychaete species that utilizes solid waste materials and recommends it as an efficient species for aquaculture solid waste management.

**Graphical abstract:**

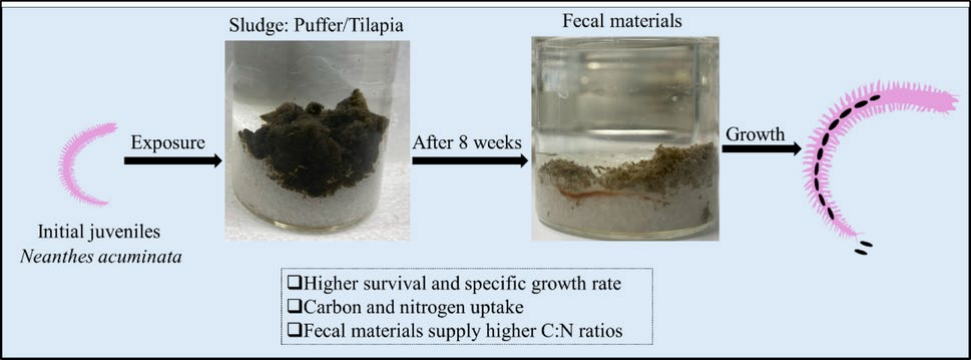

**Supplementary Information:**

The online version contains supplementary material available at 10.1007/s11356-026-37698-9.

## Introduction

Aquaculture has played a crucial role in providing essential proteins and nutrients to combat the challenges of meeting the increasing demands of a growing human population (Troell et al. 2014; FAO 2024). Globally, aquaculture production reached 130.9 million tonnes, of which 72.1% was aquatic animals, and is expected to rise by an additional 22% to meet the increasing global demand (Blanchard et al. 2017; FAO 2024). Increasing aquaculture production could lead to excessive feed supplementation and pose a significant threat to the environment by disrupting ecological balance (Bao et al. 2019; Bohnes et al. 2019; Boyd et al. 2020). Recently, technologically advanced systems have been used to manage, reuse, and recycle materials within aquaculture systems (Martins et al. 2010; Van Rijn 2013; Yogev et al. 2017). However, the challenges remain in limiting and recycling the supply of solid waste as leftover feed, along with metabolic wastes, which accumulate as particulate organic matter (i.e., OM), total dissolved solids, suspended solids, dissolved nutrients, and metabolites. Any mismanagement in releasing waste materials into the environment can cause alterations (e.g., eutrophication and increased algal blooms) in the natural environment (Camargo and Alonso 2006; Cai et al. 2013; Herath and Satoh 2015; Muziasari et al. 2016). Sustainable solid waste management protocols are still being developed; therefore, understanding is limited.

Organic sludge, a byproduct of aquaculture farms, poses significant challenges due to its unknown environmental consequences. The sharp increase in aquaculture production increases feed consumption in the aquaculture process. Consequently, a significant proportion of carbon (i.e., C) and nitrogen (i.e., N) could be accumulated within sludge, which consists of unused feed, metabolic, excretory, and respiratory wastes. Continuation of this process can result in substantially higher levels of C and N (62% of feed C and 57% of feed N) in the environment (Wang et al. 2012, 2013). The nutrient conversion rate by fish is insufficient to maintain a proper balance within aquaculture cages, often leading to significant imbalances (Wen et al. 2007). Although the Recirculating Aquaculture Systems (RAS) have recently been used in many industries, which facilitate the disposal of solid waste, reuse, and recirculation of the water within the system (Van Rijn 2013; Yogev et al. 2017); hence, managing the solid wastes has been a constraint due to the excessive production load (Luo et al. 2015). The extracted sludge contains a combination of higher nutrient and organic content, which has been estimated to be up to 40% of the total supplied feed content in a properly managed aquaculture farm (Elbeshbishy and Nakhla 2011). It demonstrates that the load generated by organic sludge could impair the ecological structure and function, resulting from environmental deterioration (Rodriguez et al. 2007). Several attempts have been made by scientists using biological monitoring in RAS; among all polychaete-based systems, some have raised the possibility of sound management of both solid and liquid wastes (Brown et al. 2011; Gómez et al. 2019). The RAS, coupled with the polychaete, could facilitate multiple ways. For instance, polychaetes can be cultured within aquaculture sludge. These raised polychaetes could be a potential food source for the cultured fish. Due to the diverse species-specific efficiencies, understanding the bio-based remediation of aquaculture sludge (e.g., remediation by bacteria, polychaetes, or algae) remains limited.

Few aquaculture systems have been established to support a circular bioeconomy through a simultaneous bio-based remediation process (Stabili et al. 2010; Nederlof et al. 2019). The deposit-feeding polychaetes have the potential to efficiently ingest and assimilate organic waste within their bodies; thus, developing an aquaculture system integrated with polychaetes could be a sustainable solution for sludge remediation, enabling the recycling and reuse of most resources used. To establish a proper circular methodology, the polychaetes’ growth, survival, reproduction, and regeneration must be assessed under sludge-exposure conditions. Few species have been examined to verify their bioremediation potential by confirming their survival, growth, organic component, and nutrient uptake capabilities in aquaculture sludge (Stabili et al. 2010; Nederlof et al. 2019; Gómez et al. 2019, 2023). In a study, Jansen et al. (2019) found that the polychaete species *Ophryotrocha* spp. could reduce organic waste, such as fish feces and uneaten feed from fish farms in Western Norway. These polychaetes could play a critical role in upcycling the nutrients and bioaccumulating proteins, lipids, and fatty acids contained in aquaculture sludge (Jerónimo et al. 2020; Malzahn et al. 2023; Anglade et al. 2023a, b).

Although some previous reports have examined the bioremediation efficiency of polychaetes, more comprehensive studies using diverse species and sludge types are needed to expand our knowledge and provide an extensive database for large-scale utilization of polychaete-based bioremediation. We chose the polychaete species *Neanthes acuminata* (Annelida: Nereididae), which has not previously been employed to examine its bioremediation potential (Ehlers 1868; Weinberg et al. 1990). This species has been ubiquitously distributed and established as playing unique ecological functions and as an essential component of marine food webs (Sutton et al. 2005; Reish et al. 2014). Their repeated reproductive capability made them a strong candidate for bioremediation of aquaculture sludge. To extend existing knowledge and understanding of the bioremediation potential of diverse polychaetes across different aquaculture sludge types, we aimed to examine nutrient and organic carbon (i.e., OC) ingestion, assimilation, and upcycling potential in *N. acuminata* across sludge types collected from puffer and tilapia fish farms. We hypothesized that *N. acuminata* individuals would be able to survive and grow across different sludge types by successfully upcycling sufficient nutrients within their bodies and could potentially supplement the fish feed, thus contributing to solid waste management. As an efficient tool for understanding trophic interactions, carbon and nitrogen stable isotope ratios (i.e., SIRs) have been consistently used to determine the relationships between organisms and their nutrient sources in aquatic systems (Peterson and Fry 1987; Fry 2006). This technique is based on the assumption that stable isotope signatures do not decay over time and thus can provide information on the diet of consumers integrated over temporal dynamics (e.g., from days to months, based on varying rates of tissue turnover; Fry and Sherr 1989). Consumer’s C SIR signatures often reflect their diet, whereas consumer’s N SIR values increase by a stepwise manner (e.g., average 2.3 to 3.4‰) relative to the basal food resources (Peterson and Fry 1987; Post 2002; McCutchan et al. 2003). The application of SIRs to understand trophic interdependence and nutrient assimilation remains limited in aquaculture ponds (Parker et al. 1989; Mahmood et al. 2016). Thus, the current study employed C and N SIRs as a tool to determine the trophic interplay of *N. acuminata* across different aquaculture sludges.

## Materials and methods

### Collection and preparation of test organisms

The sediment samples (abundant in *N. acuminata*) have been collected from Uwakai Sea in southern Ehime, Japan, every year and have been cultured at the Fisheries Technology Institute’s laboratory in Hatsukaichi, Japan, for more than a decade without modifying the characteristics of the bedrock materials. The *N. acuminata* individuals were maintained in continuously flowing, filtered, natural seawater at 20 °C, with sufficient aeration. The individuals were fed commercially available fish feed Otohime (Marubeni Nisshin Feed Co. Ltd., Aichi, Japan; feed composition: crude protein 50%, crude fat 10%, crude fiber 3%, crude ash 17%) in the culture. Juveniles of *N. acuminata* were used from the in-house laboratory culture sediment with an initial wet weight of 8.06 ± 2.91 mg (mean ± standard deviation; N = 30).

### Aquaculture sludge

The aquaculture sludge was collected separately from the recirculating systems of two different fish species, namely, puffer (i.e., *Takifugu rubripes*) and tilapia (i.e., *Oreochromis niloticus*), which were operated with 2% (e.g., 20 g/L) diluted seawater (Online Resource Supplementary Fig. S1). The fish feeds Hirame EP-F4 (i.e., Marubeni Nisshin Feed Co. Ltd., Tokyo, Japan; composition: crude protein 50%, crude fat 6%, crude fiber 2%, crude ash 17%) and Twin power (i.e., NOSAN, Yokohama, Japan; composition: crude protein 43%, crude fat 10%, crude fiber 4%, crude ash 15%) have been used in puffer and tilapia aquaculture respectively. After collection, sludges (i.e., puffer and tilapia sludge) were centrifuged (e.g., at 3000 rpm for 10–20 min) to separate the overlying water, which was then preserved immediately at 4 °C to prevent bacterial degradation. Each time, processed sludge was used in the experimental treatments for no more than 4 weeks, regardless of sludge type. For the initial characterization, water content, OM content, C and N SIRs, elemental C to N ratios, and elemental C and N contents were determined. The water content was determined by drying the sludge in the oven at 110 °C for 2 h. The total OM content in the sludge was determined by incinerating it at 550 °C for 3 h (FO310, Yamato Scientific Co., Tokyo, Japan). For the stable isotope analysis, sludge samples were collected right after centrifugation. Prior to SIRs analysis, the samples were freeze-dried and ground to a fine powder. The C and N SIRs are expressed with the following equation, where delta notation denotes the relative differences between samples and conventional standards (i.e., Vienna Peedee Belemnite (VPDB) for C and air N_2_ for N, respectively).

1 $$\updelta X=\left(\frac{{R}_{\mathrm{sample}}}{{R}_{\mathrm{standard}}} -1\right)\times 100$$

where *X* denotes the isotope of interest (e.g., ^13^C) and *R* denotes the relative abundance of stable isotopes of the respective element in samples and standards (e.g., ^13^C/^12^C).

The C (δ^13^C) and N (δ^15^N) SIRs were determined using mass spectrometers coupled with elemental analyzers (Thermo Fisher Scientific Delta V Advantage Isotope Ratio MS, Bremen, Germany). Known standard delta values of L-alanine were analyzed at least every 10 runs to confirm reproducibility (i.e., < 0.15 ‰ for both N and C SIRs).

The protein content was obtained from the nitrogen content by multiplying by the conventional protein conversion factor (i.e., 6.25) following Eq. 2 (Fang et al. 2016).

2 $$\text{Crude protein }(\mathrm{\%})=\text{Nitrogen content }(\mathrm{mg}/\mathrm{mg})\times \mathrm{F}\times 100$$

where nitrogen content (mg/mg) denotes the amount of elemental nitrogen content within the samples used, and F denotes the conversion factor.

### Growth and survival assay

After confirming the initial survival and growth in different salinity and sludge (Online Resource Supplementary Text S1-S2; Online Resource Supplementary Fig. S2; Online Resource Supplementary Table S1), the microcosms were developed in puffer and tilapia sludge separately with a negative control without feeding using a 150 mL vial where 50 g of silica (e.g., as a substrate), 100 mL seawater (i.e., 20 g/L SW), and 1.5 to 3.5 g puffer and tilapia sludge/week (i.e., 3 to 7% of the inert silica) were used with aeration in an incubator at 20 °C for examining survival and growth for 8 weeks (*N* = 10 organisms for each treatment). Each treatment type comprised 10 independent replicate experimental units, with one organism per vial. The amount of sludge was measured using a weighing scale and carefully placed on the silica surface in each vial, avoiding disturbance as much as possible. The overlying water in each vial was replaced weekly. Individual survival and body weight were measured every week. Surviving individuals were divided by the initial number for each sludge treatment type to calculate the weekly survival rate. The weight gain was measured individually using Eq. 3 and was thus not adjusted for the survival ratio. The specific growth rate (i.e., SGR) was calculated using the following Eq. 4 (Anglade et al. 2023a). All weight parameters were measured on a wet-weight basis.

3 $$\text{Weight gain}={\text{Final weight }}_{(\text{survived individual})}-{\text{Initial weight }}_{(\text{average pooled replicates})}$$

where the final weight was considered for individual survived *N. acuminata* juveniles every week, and the initial weight denotes the pooled average of all individuals that were measured at the initial stage.

4 $$\text{SGR }(\mathrm{\%})=\left(\frac{\mathrm{ln}\left({W}_{\mathrm{t}}\right)-\mathrm{ln}\left({W}_{0}\right)}{\mathrm{t}}\right)\times 100$$

where, *W*_t_ is the final wet weight (mg), *W*_0_ is the initial wet weight (mg), and *t* is the duration of the exposure trial.

### Nutrient uptake assay

We measured the C and N SIRs of *N. acuminata* juveniles as a proxy to investigate nutrient assimilation and upcycling within their bodies after exposure to the sludges. For this purpose, two sets of microcosms were developed using different sludge types and maintained for 10 days under similar experimental conditions as described in the above “Growth and survival assay” sub-section. The test specimens for SIRs were collected at 0 (e.g., *N. acuminata* juveniles from culture as initial measurement), 3 (e.g., *N. acuminata* juveniles), and 10 days (e.g., *N. acuminata* juveniles and fecal materials). The sample processing details were described in the “Online Resource Supplementary Text S3.” The N and C SIRs were conducted following the protocols described in the above subsection of “Aquaculture sludge.” Elemental ratios were determined based on the N and C content obtained from each analyzed sample in “mg/mg dry weight.” We measured C and N uptake ratios using Eq. 5 (Anglade et al. 2023b).

5 $$\text{Uptake }(\mathrm{\%})=\left(\frac{{\text{Amount }(Na)}_{\text{contained }(\mathrm{initial})} - {\text{Amount }(Na)}_{\mathrm{contained}(\mathrm{after})}}{{\text{Amount }(\mathrm{sludge})}_{\text{ supplied}}}\right)\times 100$$

The contained amounts of “initial” and “after” represent the amounts of C and N within *N. acuminata* (mg/mg) before and after the experiment (i.e., 3 and 10 days), respectively. The supplied amount represents the C and N (mg/mg) contents existing in the sludge. We measured OC assimilation using Bianchi’s model (Bianchi 2007) as follows:

6 $$\text{OC assimilation }(\mathrm{\%})=\left(\frac{{{\updelta }^{13}\mathrm{C}}_{Na} - {{\updelta }^{13}\mathrm{C}}_{\mathrm{standard}}}{{\updelta }^{13}{\mathrm{C}}_{\mathrm{initial}} - {{\updelta }^{13}\mathrm{C}}_{\mathrm{standard}}}\right)\times 100$$

where, δ^13^C_Na_ denotes the final isotopic composition of *N. acuminata* juveniles, δ^13^C_initial_ denotes the isotopic composition of sludge, and δ^13^C_standard_ represents the isotopic composition of *N. acuminata* juveniles in the wild. In this study, *N. acuminata* juveniles from the laboratory culture were considered the δ^13^C standard, as they have been maintained in the laboratory for many years in recirculating natural seawater without manipulation of their sedimentary characteristics since collection from their original habitat (see “Collection and preparation of test organisms” subsection in “Materials and methods”).

### Statistical analysis

We examined the survival, growth, SGR, and nutrient uptake efficiency of *N. acuminata* juveniles across different sludge types by constructing either generalized linear mixed models (GLMMs) or generalized linear models (GLMs). First, we developed GLMMs for growth and SGR separately to examine whether they differ among treatment types (i.e., negative control, puffer, and tilapia sludge) and observation weeks by considering growth and SGR as response variables; treatment types, observation weeks, and their interactions as explanatory variables, and number of individuals survived every week as random factor with Gaussian error distribution family. Additionally, we tested whether growth and SGR patterns differ between sludge types by excluding the negative control from the previous model. For this purpose, we developed GLMMs by considering growth and SGR as response variables, and treatment types (i.e., puffer and tilapia sludge), observation weeks, and their interactions as explanatory variables. The number of individuals surviving each week was treated as a random factor with a Gaussian error distribution. Then, we tested whether C and N SIRs of *N. acuminata* juveniles differed after exposure to the sludge types by separately (i.e., sludge type) developing GLMs with C and N SIRs as response variables and treatment type as an explanatory variable (Gaussian error distribution). To examine how the C to N ratios differed before and after exposure scenarios, GLMs were separately developed with C:N ratios as response variables and sample types (e.g., initial and exposed *N. acuminata* juveniles, initial sludge, and fecal materials after 10 days of exposure) as explanatory variables with a Gaussian error distribution. To examine nutrient uptake rates in *N. acuminata* juveniles during exposure to different sludge types, GLMs were constructed with C and N uptake rates and OC assimilation as response variables, with sludge type as a fixed factor and assuming a Gaussian error distribution.

All statistical analyses were performed in R (version 4.3.2, R Core Team 2023). The GL(M)Ms adopted a Gaussian error distribution. The likelihood ratio tests were used to assess the impact of the variables of interest by sequentially comparing models that excluded them. The multiple comparisons among the variables in GLM(M)s were carried out using the ‘multcomp’ package. The statistical significance level was set at *p* = 0.05, with Bonferroni corrections applied to group comparisons.

## Results

### Sludge characterization

The amounts of C and N differed significantly between sludge types, with higher contents in tilapia (i.e., *O. niloticus*). The C and N SIRs also differed between sludge types, with a higher ratio in puffer (i.e., *T. rubripes*) sludge (15.6 ± 0.06 and −16.3 ± 0.11 (mean ± standard deviation) mg/mg dry weight for N and C, respectively). The C:N ratios did not differ between sludge types (Table 1). The puffer and tilapia sludge types are characterized by 86.8 to 89.2% of water, with a notable difference in OM content, in which tilapia sludge contained 73.1% of OM and was higher among the sludge types (Table 1). Crude protein content was significantly higher in tilapia sludge than in puffer sludge.

**Table 1:** Characterization of aquaculture sludge (mean ± standard deviation)

Characteristics	Puffer sludge	Tilapia sludge
Water content (%)	86.9 ± 0.06	89.2 ± 0.33
OM (%)	40.7 ± 0.06	73.1 ± 0.33
Nitrogen stable isotope ratios (‰)	15.5 ± 0.06	3.8 ± 0.20
Carbon stable isotope ratios (‰)	−16.3 ± 0.11	−23.2 ± 0.12
N (mg/mg dry weight)	0.02 ± 0.00	0.05 ± 0.00
C (mg/mg dry weight)	0.2 ± 0.00	0.4 ± 0.01
C:N ratio	6.9 ± 0.09	6.4 ± 0.22
Protein content (%)	15.0 ± 0.42	32.9 ± 1.35

### Survival and growth within sludge

**Fig. 1:**
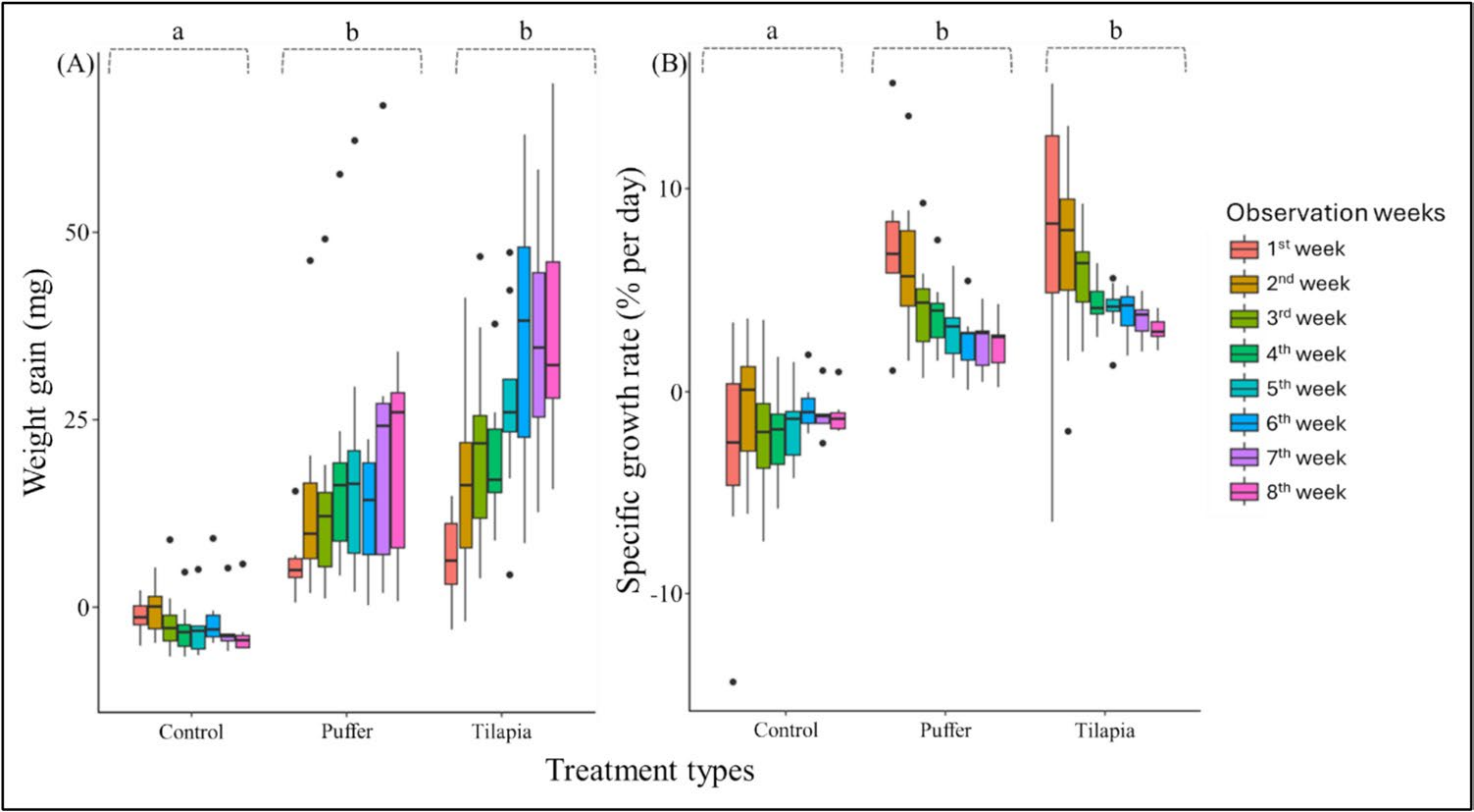
Weight gain pattern of *N. acuminata* in two different sludge types. Comparisons of the growth (**A**) and SGR (**B**) patterns across different aquaculture sludge types compared with the negative control. Alphabetical small letters denote the results of multiple comparisons based on generalized linear mixed models; those with different letters were statistically distinguishable (*p* < 0.05)

The survival of *N. acuminata* juveniles did not differ between sludge treatments. It ranged between 80 and 90% (Table 2). In contrast, the percentage was lower (approximately 60%) in the negative control, which did not receive feeding during the 8-week exposure period. The initial wet weight of *N. acuminata* juveniles was 7.9 to 8.2 mg (Table 2). The growth and SGR patterns differed among treatment types, with the SGR ranging from −0.72 to −2.75%, 2.2 to 6.69%, and 3.02 to 7.54% day^−1^ in negative control (no feeding), puffer, and tilapia sludge, respectively (GLMM: *p* < 0.05; Fig. 1; Online Resource Supplementary Table S2a, b, S3). The growth and SGR patterns interactively changed across treatment types (i.e., negative control, puffer, and tilapia sludge) and experimental weeks (Fig. 1; Online Resource Supplementary Table S2a, b). However, the weight gain and SGR in the tilapia sludge were significantly higher than those of the puffer when comparing them without considering the negative control in the model (Table 2; Online Resource Supplementary Table S2c, d). On average, the weight gain of *N. acuminata* juveniles was 5.4 to 26.7 mg and 6.7 to 40.3 mg across experimental weeks in puffer and tilapia sludge, respectively.

**Table 2:** A table summarizes the initial and final weight (mean ± standard deviations) and survival rate of *N. acuminata* juveniles in the 8-week growth assay across different sludge treatment levels

Week	Control/no feeding			Puffer sludge			Tilapia sludge		
	Initial weight (mg)	Final weight (mg)	Survival rate (%)	Initial weight (mg)	Final weight (mg)	Survival rate (%)	Initial weight (mg)	Final weight (mg)	Survival rate (%)
1st	8.2 ± 3.17^(10)^	7.0 ± 2.08^(10)^	100%	8.1 ± 2.89^(10)^	13.5 ± 4.15^(10)^	100%	7.9 ± 2.64^(10)^	14.6 ± 5.51^(10)^	100%
2nd		7.1 ± 2.91^(10)^	100%		22.4 ± 12.56^(9)^	90%		23.8 ± 11.94^(10)^	100%
3rd		6.4 ± 4.17^(10)^	100%		22.3 ± 13.43^(9)^	90%		29.8 ± 12.29^(10)^	100%
4th		5.5 ± 3.19^(9)^	90%		26.1 ± 15.42^(9)^	90%		27.3 ± 7.95^(10)^	100%
5th		5.2 ± 3.42^(8)^	80%		28.1 ± 17.02^(9)^	90%		34.8 ± 12.03 ^(9)^	90%
6th		7.2 ± 4.78^(7)^	70%		27.6 ± 19.73^(9)^	90%		43.4 ± 16.57^(9)^	90%
7th		5.5 ± 3.68^(6)^	60%		31.2 ± 19.37^(8)^	80%		48.2 ± 19.68^(9)^	90%
8th		5.3 ± 3.96^(6)^	60%		34.9 ± 23.06^(8)^	80%		45.3 ± 15.37^(9)^	90%

*Bracketed superscripts denote the number of replicates

#### Isotopic signatures of *N. acuminata* within sludge

**Fig. 2:**
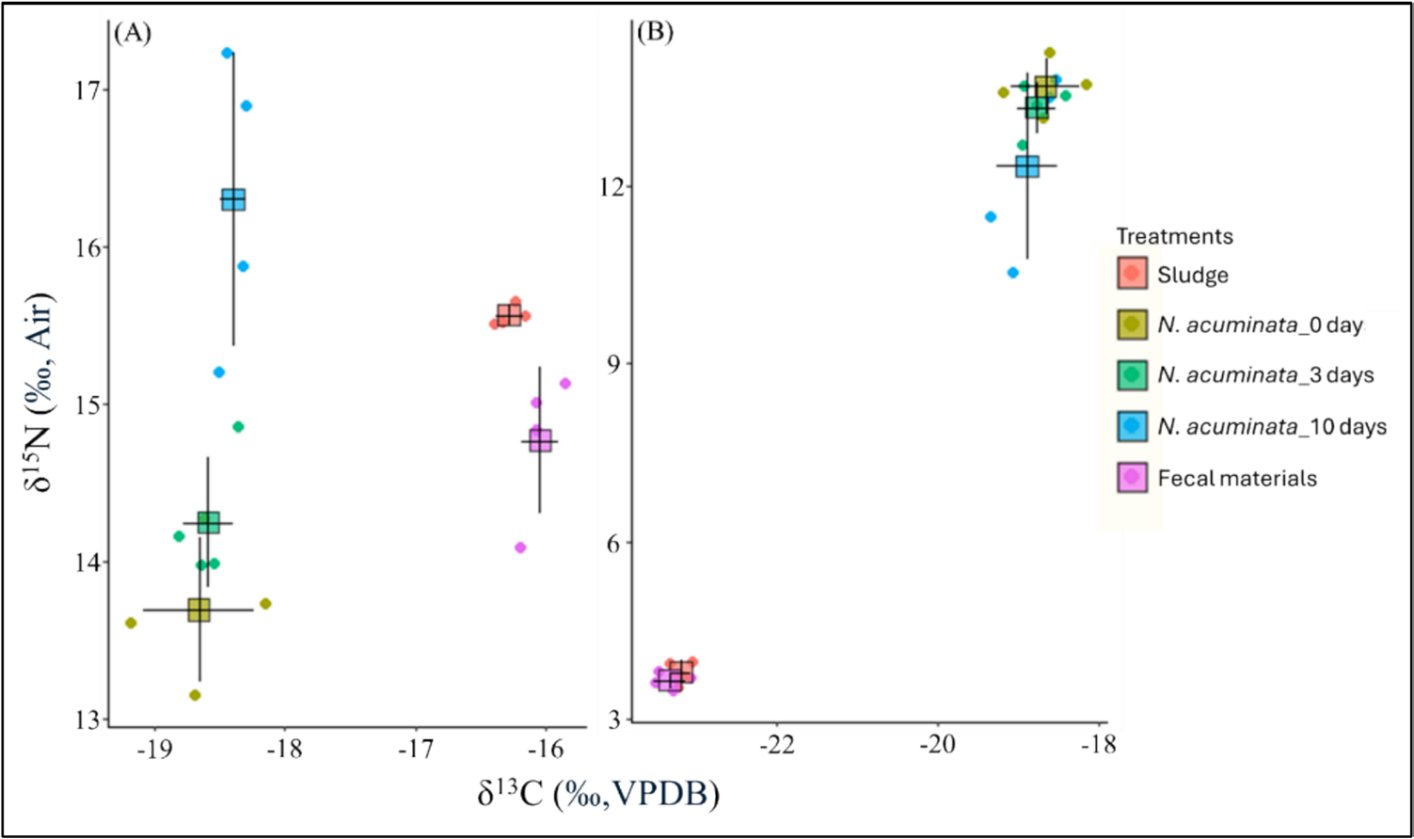
Biplots of carbon and nitrogen stable isotope ratios** (**mean ± standard deviations) for *N. acuminata* in puffer (**A**) and tilapia (**B**) sludges. The plots also include the carbon and nitrogen stable isotope ratios of the provided sludge and fecal materials, with the initial pattern of *N. acuminata*

At the beginning of the experiment, the initial N and C SIRs of *N. acuminata* individuals and sludge types were significantly different (Fig. 2; Online Resource Supplementary Table S4). The N SIRs of *N. acuminata* showed significant enrichment (i.e., 16.30 ± 0.93 ‰; mean ± standard deviation) after 10 days of exposure to the puffer sludge (Fig. 2; Online Resource Supplementary Table S4-S6), while N SIRs were depleted (12.35 ± 1.58 ‰; mean ± standard deviation) at the end of exposure in tilapia sludge (Fig. 2; Online Resource Supplementary Table S5-S6). Although the juvenile’s C SIRs did not significantly differ after exposure, they tended to change toward the sludge isotopic ratios (Fig. 2; Online Resource Supplementary Table S4-S6). The changes in N and C SIRs of *N. acuminata* individuals after exposure followed the isotopic signatures of their food sources, as well as the types of sludge in all cases. On the other hand, fecal materials of *N. acuminata* individuals showed a statistically similar signature (e.g., 14.77 ± 0.46 and −16.04 ± 0.14 ‰; mean ± standard deviation N and C SIRs respectively for puffer and 3.65 ± 0.13 and −23.34 ± 0.2 ‰; mean ± standard deviation N and C SIRs respectively for tilapia) within respective sludge types (Fig. 2; Table 1, Online Resource Supplementary Table S4-S6).

**Fig. 3:**
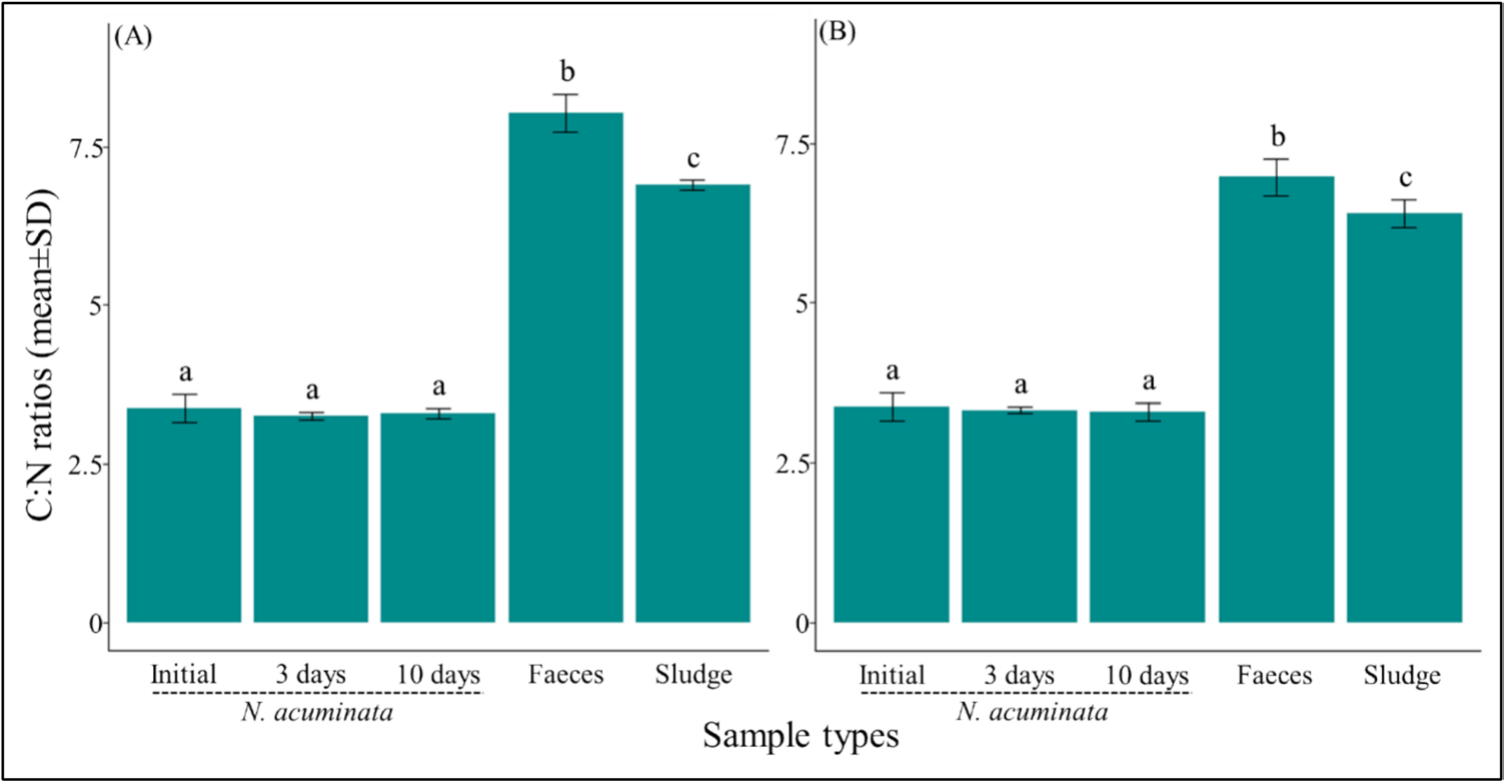
Mean (± standard deviations) carbon-to-nitrogen ratios of sludge, *N. acuminata* across the sludge exposure periods, and fecal materials in Puffer (**A**) and Tilapia (**B**) sludges. The lowercase letters denote the results of multiple comparisons based on generalized linear models; those with different letters were statistically distinguishable (*p* < 0.05) within each sludge exposure type

In both sludge types, the C:N ratio of *N. acuminata* juveniles did not show any significant difference relative to the initial level toward the exposure durations (e.g., 3 days or 10 days). However, the C:N ratio showed substantial differences among *N. acuminata* juveniles, fecal material, and sludge (Fig. 3; Online Resource Supplementary Table S7). The highest reported C:N ratio of 8.03 ± 0.33 (mean ± standard deviation) was obtained in fecal materials of puffer sludge, which was significantly different than that of tilapia sludge (i.e., fecal materials > sludge types). Regardless of sludge type, *N. acuminata* juveniles showed increasing trends in C and N content relative to initial levels, although these changes were not statistically significant. The C and N contents did not increase significantly with the extended exposure duration (Online Resource Supplementary Table S8).

### Nutrient uptake within sludge

**Fig. 4:**
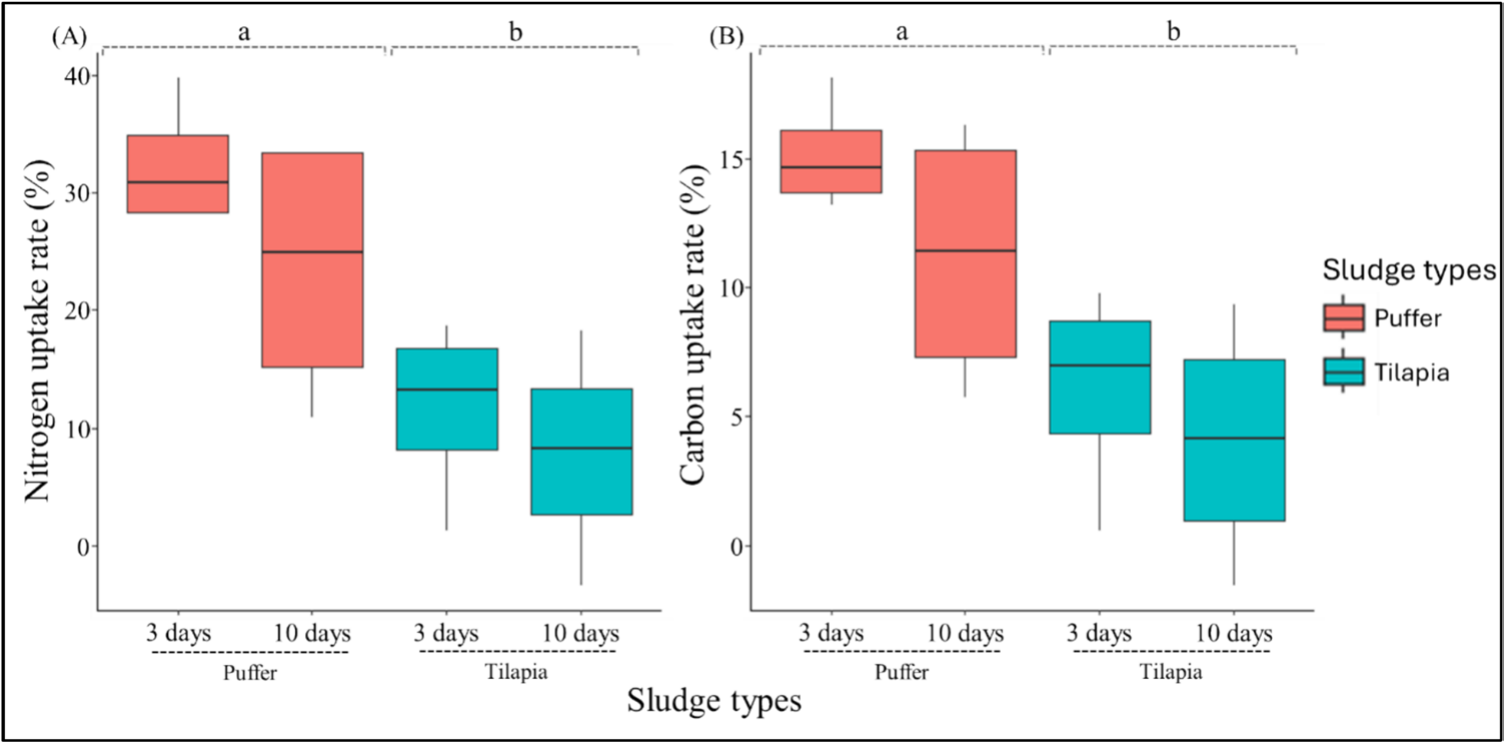
Nitrogen (**A**) and carbon (**B**) uptake rates by *N. acuminata*. Nitrogen and carbon uptake rates during the sludge exposure period. The lowercase letters denote the results of multiple comparisons based on generalized linear models; those with different letters were statistically distinguishable (*p* < 0.05)

The juveniles fed on puffer sludge showed a significantly higher N uptake rate than those in tilapia sludge (GLM: *p* < 0.05; Online Resource Supplementary Table S9a). The N was accumulated at a rate of 32.42 ± 5.44% (mean ± standard deviation) in 3 days and was subsequently slowed down in 10 days (23.58 ± 11.64%; mean ± standard deviation; Fig. 4) in juveniles exposed to puffer sludge. The C uptake ratios followed a similar trend to N and showed a significant difference between sludge types (GLM: *p* < 0.05; Online Resource Supplementary Table S9b). On the other hand, the OC assimilation rate within the *N. acuminata* body was not significantly different between sludge types. However, they showed maximum OC assimilation of 11.08 ± 7.93% and 5.04 ± 8.45% (mean ± standard deviation) in 10 days within puffer and tilapia, respectively (Online Resource Supplementary Fig. S3). No noted differences were reported between sludge types (GLM: *p* > 0.05; Online Resource Supplementary Table S9c). We found a significant difference in OC between exposure days (GLM: *p* < 0.05) in the puffer sludge treatment, but not in the tilapia sludge (i.e., 10 days > 3 days).

## Discussion

Developing an environment-friendly and sustainable sludge management technique is urgently demanded to manage the excessive amount of solid waste produced daily in the aquaculture industry. For the first time, we investigated whether the polychaete *N. acuminata* can survive and grow in two types of sludge (puffer and tilapia). We examined its active utilization of available nutrients in the sludge. The polychaete juveniles acted as a functional transformer of sludge nutrients, efficiently assimilating them in both types of sludge, thereby demonstrating their capability as a competent remediation tool for aquaculture solid waste management. This study suggests that *N. acuminata* juveniles can be effectively utilized in the sound management of aquaculture solid waste by incorporating them into recirculating systems as a biological solution for removing OM and nutrients.

The juveniles of *N. acuminata* exhibited successful survival relative to the negative control without feeding, indicating their ability to thrive in various types of sludge. The higher survival rate in sludge treatments indicated a more abundant nutritional supply than in the negative control, where a lower survival rate (i.e., 60%) was observed, possibly due to starvation and predation by other individuals (Yousefi-Garakouei et al. 2019). Although it is obvious that survival would be affected without feeding, the higher survival rate indicates *N. acuminata’s* capability and suitability across different aquaculture sludge types. Olive et al. (2006) reported a 93.3% survival rate for the deposit-feeding worm *Arenicola marina* on foodstuffs such as brewery yeast and fish farm waste. In contrast, *Hediste diversicolor* showed a significantly lower survival rate in another study reported by Jerónimo et al. (2021). However, higher survival rates across sludge experiments vary with experimental conditions, habitat quality, and nutrient availability, as frequently reported in studies (Bischoff 2007; Gómez et al. 2023). Some previous studies controlled the exchange of overlying water in experimental setups or vials, arguing for improved habitat quality for deposit feeders (Honda and Kikuchi 2002; Gómez et al. 2023). Our study was limited to weekly water exchange and confirmed a minimum survival rate of 80% for both sludge types. The interactive effect of sludge treatments on weight gain and SGR across experimental weeks indicated that they could grow efficiently in the sludge. The average SGR was 3.89% and 4.87% day^−1^ in puffer and tilapia sludge, respectively, which are significantly higher than those reported in many studies (Yearsley et al. 2011; Hu et al. 2021). Pajand et al. (2017) reported SGRs of 3.40% and 3.39% day^−1^ for *Nereis diversicolor* in sturgeon fish feces and feed waste, respectively, for approximately 8 weeks. Honda and Kikuchi (2002) reported 1.66% day^−1^ SGR in *Perinereis nuntia vallata* after culturing within flounder feces for 15 days. Knowledge and understanding of growth rate trends within different sludge types are mixed due to the diversity of experimental designs, which vary in terms of sludge and polychaete species. Several studies have compared the SGR of deposit feeders after exposure and culturing them in sludge at different feeding levels but have given little consideration to their efficiency when exposed to sludge from various fish species (Yearsley et al. 2011; Hu et al. 2021). The current study revealed a significantly higher growth and SGR, indicating efficient energy and nutrient utilization by *N. acuminata*. Because different species may have unique resource-use efficiencies (Fauchald and Peter 1979), we report that *N. acuminata* juveniles can grow at a notably higher rate.

The growth rate of *N. acuminata* individuals was relatively higher in tilapia sludge and was statistically different from that of individuals reared on puffer sludge. Although the current study did not include a control replicate using commercial fish feed, similar species showed a comparable weight-gain pattern in a study by Alam and Ito et al. (2025), which used commercial fish feed (e.g., Otohime, NOSAN, Yokohama, Japan). Thus, the weight gain ratios in the current findings could be considered a regular trend associated with sufficient nutrient uptake. Feed quality, feed quantity, feed availability, genetic variability, size class, experimental design, and animal density have been recognized as influential factors affecting the growth rate (Kurihara 1983; Scaps 2002; Yousefi-Garakouei et al. 2019; Jerónimo et al. 2020). Yousefi-Garakouei et al. (2019) demonstrated the bioremediation efficiency of *N. diversicolor* in a rearing experiment from wastewater-integrated aquaculture systems with rainbow trout (i.e., *Oncorhynchus mykiss*). They found effective weight gain under density-dependent conditions. Our study did not aim to examine density-dependent variability, but rather to determine how *N. acuminata* juveniles interact with different types of sludge. The initial juveniles used in the experiment were comparable across sludge treatments, with a range of 7.89 ± 2.64 to 8.1 ± 2.89 mg (mean ± standard deviation), which rejects the hypothesis of inconsistencies due to the mixture of different size classes within similar treatment types. Thus, the significantly higher weight gain and SGR patterns in tilapia sludge could have originated solely from the higher OM and crude protein content (Table 1). The significantly higher organic matter content may affect the growth ratios of individuals reared on tilapia sludge, a finding also evident in previous studies (Bridges et al. 1994; Bridges 1996). The amount of fecal cast production among the sludge treatments did not differ significantly based on visual observation; thus, individuals in the puffer sludge treatment did not exhibit any change in behavioral traits, as predicted by optimal foraging theory (Pyke 2019). A study by Taghon and Jumars (1984) found variable feeding rates among three deposit-feeding polychaete species, with higher ingestion rates when sediments contained lower protein content. Although omnivorous, the family Nereididae (i.e., *N. acuminata*) can use available resources in sediments; however, they can switch feeding modes based on those resources, enabling efficient use of available resources (Galasso et al. 2018). In the present study, *N. acuminata* utilized resources regardless of sludge type to meet metabolic demand.

The prediction that *N. acuminata* would successfully uptake the nutrients from the sludge was supported. *N. acuminata* showed higher N and C uptake rates across 10-day exposure periods, consistent with previously reported findings (Honda and Kikuchi 2002; Fang et al. 2016). There was no difference in day intervals between the two sludge types, indicating that the requirement for deposit-feeding polychaetes maintains a similar trend when exposed to sludge. However, it may vary based on feeding activity and metabolic mechanisms (Nederlof et al. 2019). The sludge-exposed juveniles showed clear differences in C and N content compared to the initial individuals. This supported the earlier-mentioned hypothesis regarding the opportunistic feeding strategy and nutrient utilization of deposit feeders (Galasso et al. 2018). The findings of the current study indicated differences between the two sludges, with *N. acuminata* showing a relatively higher degree of C and N assimilation from puffer sludge. The *N. acuminata* juveniles efficiently acquired C and N contents in both sludge types, as reported for other species, such as *Abarenicola pusilla* and *H. diversicolor* (Gómez et al. 2019; Anglade et al. 2023a, b). The leftover feed, fish fecal materials, and sludge aging could affect the quality and composition of the sludge (Anglade et al. 2023b). Nederlof et al. (2019) demonstrated that nutrient requirements for *Capitella* sp. and *Ophryotrocha craigsmithi* from the salmon-driven integrated multi-trophic aquaculture were 5 to 26 mg C and from 2 to 6 mg N g^−1^ ash-free dry weight per day. The sludges used in the current study were collected on a similar occasion and processed, maintaining a similar protocol. Thus, sludge collection occasion, process, and/or aging may not have a significant influence on the higher assimilation in puffer sludge. The assimilated C and N contents in *N. acuminata* did not differ significantly after a 10-day exposure period and were comparable across sludge types (Online supplementary Resource S8). The C and N content of the sludge might act as a driving factor. The higher nutrient content in tilapia sludge may have contributed to reducing the uptake rate in Eq. 5, which underestimated the uptake rate for individuals reared on tilapia sludge. However, the current data may not be sufficient to elucidate the detailed mechanisms underlying the trend in C and N assimilation across sludge types. Thus, we report the successful nutrient uptake ability of *N. acuminata* from both sludges in the current study. Future experiments that include additional parameters for sludge and exposed polychaete individuals (e.g., biochemical characterizations and parameters related to water-layer chemistry) could further clarify the mechanisms.

Few studies have examined diet-related nutrient shifts in annelids; Schmidt et al. (1999) found a nutrient-oriented shift in N SIRs towards the diet within 10 days. Moreover, the diet-tissue isotopic discrimination factor can be achieved within a few days (e.g., 2.5 days; Dubois et al. 2007; Gamboa-Delgado 2022). Thus, exposure assays to determine nutrient uptake were conducted over 10 days. Despite having fewer reports of diet-tissue isotopic shifts, a potential gap exists in the literature on marine polychaetes. More specifically, no studies have reported a diet-related shift in *N. acuminata*. Thus, we chose the time frame and intervals to determine the diet-related isotopic shift in body tissues of *N. acuminata* following sludge exposure in the current study. The N SIRs of *N. acuminata* in puffer sludge were significantly enriched after 3- and 10-day exposure relative to the initial individuals, which supported the higher N accumulation. The C SIRs did not reflect the C content gain in our observation, which could be explained as follows. First, the initial C SIRs reflect the isotopic signature of *N. acuminata* in laboratory culture, which is qualitatively different than that under experimental conditions regarding carbon sources. Second, C SIRs of sludge types were different which led to the increasing or decreasing trend toward sludge isotopic signatures after exposure (Fig. 2). The ingested C in the body could be solely utilized for metabolic processes rather somatic development in a rate where the C SIRs could not be detected toward sludge signatures (Pajand et al. 2017; Anglade et al. 2023b). A longer experimental duration could affect the C SIRs detected across the range of sludge types, which was a limitation of the current study. The alternative hypothesis is that limited uptake of detectable C sources in the sludge may have resulted in a lesser degree of change in C isotopic ratios. The C and N gains from aquaculture sludge might depend on feeding ecology (e.g., feeding traits, ingestion capacity, and ingestion rate; Anglade et al. 2024).

We were unable to detect any change in the C:N ratio of *N. acuminata* after exposure to sludge. However, significant changes were observed in the fecal materials after 10-day exposure compared to the sludge, suggesting a possible degree of change in C and N content in the sludge by *N. acuminata* (Fig. 3). Although the sludge characteristics showed a significant difference in C contents (Table 1), with a higher amount in tilapia, this did not reflect in the C:N ratios. The following mechanisms are attributed to the higher C:N ratios of fecal materials in both sludge exposure scenarios. Under non-limiting C and N, *N. acuminata* can use resources for growth and channel them into lipid synthesis and storage (García-Alonso et al. 2008). Increasing C:N ratios in fecal materials could facilitate the supply of OC resources in higher quantities. Consistent with previous findings, we observed higher OC assimilation in both sludge types after 10 days of exposure, supporting the mechanism described above (Fang et al. 2016; Wang et al. 2019). This process involves continuous repetition within the sludge, which could improve the material quality. Individuals would be restricted from accessing resources within the sludge because others could benefit from them, as C is usually used for energy gain and N for growth (Lopez and Levinton 1987). These individuals, reared on puffer and tilapia aquaculture sludge, could be repeatedly utilized as feed supplements for fish culture. Thus, RAS coupled with *N. acuminata* could aid in the simultaneous, efficient management of solid waste generated by land-based aquaculture industries. Moreover, the successful occurrence of secondary production through reproduction in aquaculture sludge has been a potential constraint that has been lacking for years. The current study detected the yolk content during the 8-week growth and survival experiment; however, due to the incomplete and unsuccessful reproduction, the data were not included in this research. Therefore, we recommend that *N. acuminata* can be an efficient extractive species for managing aquaculture solid waste.

## Conclusions

In summary, this study demonstrated the bioremediation efficiency of *N. acuminata* species using two commonly practiced aquaculture sludges for the first time. The results of the current study confirmed the hypothesis that *N. acuminata* can be grown successfully using these sludges as a potential nutrient source, demonstrating their efficiency in transforming and assimilating nutrients into body tissues. Furthermore, the puffer and tilapia sludge could be utilized as substrates and food sources, allowing *N. acuminata* juveniles to survive, thrive, and grow by taking up sufficient nutrients, as effectively measured using the C and N SIR approach. The higher upcycling efficiency of the resources present in sludge makes them ideal candidate species for bioremediation. The optimal C:N ratio is crucial for understanding N cycling and NH_4_^+^ assimilation in relation to bacterial growth efficiency (Robinson et al. 2019). Relatively higher C:N ratios in the eliminated fecal materials exhibited their ability to transform and improve the basal materials within the sludge environment. The integration of *N. acuminata* culture into the RAS system could successfully facilitate sustainable, recirculating management of aquaculture solid waste. This study was limited to the 10-day exposure design to determine the trophic dynamic between sludge and *N. acuminata* juveniles, which made it difficult to detect the carbon signature after the exposure period. A long exposure period may enable relatively precise detection of all isotopic signatures due to the nutrient assimilations. Future studies, including the simultaneous analysis of survival, growth, nutrient uptake, and successful reproduction within sludge, could provide a more comprehensive and sustainable approach to constructing a self-directed, automated system for managing solid and liquid waste in aquaculture.

## Supplementary Information

Below is the link to the electronic supplementary material.

ESM 1 DOCX (1.40 MB)

## Data Availability

The datasets used and analyzed during the current study are available from the corresponding author on reasonable request.
