# Supplementary material for: Nutrient assimilation from puffer and tilapia aquaculture sludge by marine polychaete Neanthes acuminata (Ehlers, 1868): a way forward to solid waste management
Source: Environ Sci Pollut Res Int. 2026 Apr 2;33(14):6509–21. doi: 10.1007/s11356-026-37698-9 (PMC13124810; doi:10.1007/s11356-026-37698-9)
Supplement: Supplementary file 1 — DOCX (1.40 MB) [file 11356_2026_37698_MOESM1_ESM.docx]

**Supplementary Information**

Nutrient assimilation from Puffer and Tilapia aquaculture sludge by marine polychaete *Neanthes acuminata* (Ehlers, 1868): a way forward to solid waste management

Md. Khorshed Alam ^a,§^, Mana Ito ^a,§^, Hiroaki Shiraishi ^b^, Akira Ohtani ^c^, Tomohiro Akagi ^d^, Yoshinobu Yamagiwa ^d^, Katsutoshi Ito ^a,*^

^a^ Environmental Conservation Division, Environment and Fisheries Applied Research Department, Fisheries Technology Institute, Japan Fisheries Research and Education Agency, 2-17-5 Maruishi, Hatsukaichi, Hiroshima 739-0452, Japan

^b^ Technical Research Institute, Okumura Corporation, 387 Ohsuna, Tsukuba 300-2612, Japan

^c^ New Business Development Department, Okumura Corporation, Marunuochi JP Tower 22F, Chiyoda-ku, Tokyo 100-7022, Japan

^d^ Industrial Technology Center of Wakayama Prefecture, 60 Ogura, Wakayama 649–6261, Japan

“*” Corresponding author (Katsutoshi Ito: ito_katsutoshi15@fra.go.jp)

“§” Md. Khorshed Alam and Mana Ito contributed equally to this work

**Supplementary text**

**Materials and methods**

**Online Resource Supplementary Text S1**

**Survival assay across different salinity dilutions**

To examine the effects of different salinity ratios used in recirculating aquaculture system (i.e., RAS), the survival ability of *Neanthes acuminata* was tested by culturing the healthy juveniles in 50 mL vial with 10 g of silica (e.g., as a substrate) and 40 mL sea water with different salinity gradients (i.e., 0, 5, 1, 15, 20, 25 g/L dilutions; N = 5 at each dilution) for 72 hours (Online Resource Supplementary Table S1).

**Online Resource Supplementary Text S2**

**Initial growth and survival assay**

Before proceeding to the final growth and survival experiment, a 7-day pre-test was conducted to verify the suitability of *N. acuminata* juveniles within sludge. For that, we examined the survival and growth of *N. acuminata* juveniles by developing a microcosms with multiple replicates (N = 8 and 10 for with and without feeding, respectively) by culturing them in 110 mL vial where 50 g of silica (e.g., as a substrate), 100 mL sea water (i.e., SW; e.g., 20 g/L salinity). The 1.5 g puffer sludge/week (i.e., 3% of the inert silica) was used with aeration in an incubator at 20 °C. Although the initial observation was limited to puffer sludge, we confirmed the survival and growth rate of *N. acuminata* juveniles (Online Resource Supplementary Fig. S2). We compared growth patterns in seawater and 20 g/L diluted seawater between treatments with and without sludge to examine *N. acuminata* growth. Feeding treatments differed between the two seawater types. In contrast, no differences were observed across seawater dilutions, supporting the use of 20 g/L diluted seawater for subsequent experimental procedures (Online Resource Supplementary Fig. S2).

**Online Resource Supplementary Text S3**

**Nutrient uptake assay**

Fecal materials were considered a proxy for the remaining OM relative to the initial sludge content, as they are transformation products and are assumed to be added at regular intervals within the sludge environment. After exposure, *N. acuminata* juveniles were left in vials containing inert silica and filtered seawater for 48 hours to defecate. After that, individuals were checked under a microscope to ensure no fecal material was present in the gut, collected in individual vials after being rinsed with water, preserved in liquid N_2_ to prevent further degradation, and kept frozen at -80 °C for later processing. After that, *N. acuminata* samples were treated for lipid extraction by repeated Chloroform-Methanol (2:1 V/V) treatment to remove the confounding effect of C and N SIRs. The preserved samples were freeze-dried, ground into a fine powder, and analysed using a protocol similar to that described in the “Aquaculture sludge” sub-section. Individual samples were collected for each replicate of *N. acuminata*; conversely, fecal material was collected as pooled samples for each treatment and day to ensure sufficient amounts, and these samples were analysed in multiple replicates to determine elemental ratios. Fecal materials were collected, freeze-dried, and ground to a fine powder prior to the analysis. At least four replicate samples (N = 4) were analysed for each treatment type on different days and sludge types.

**Online Resource
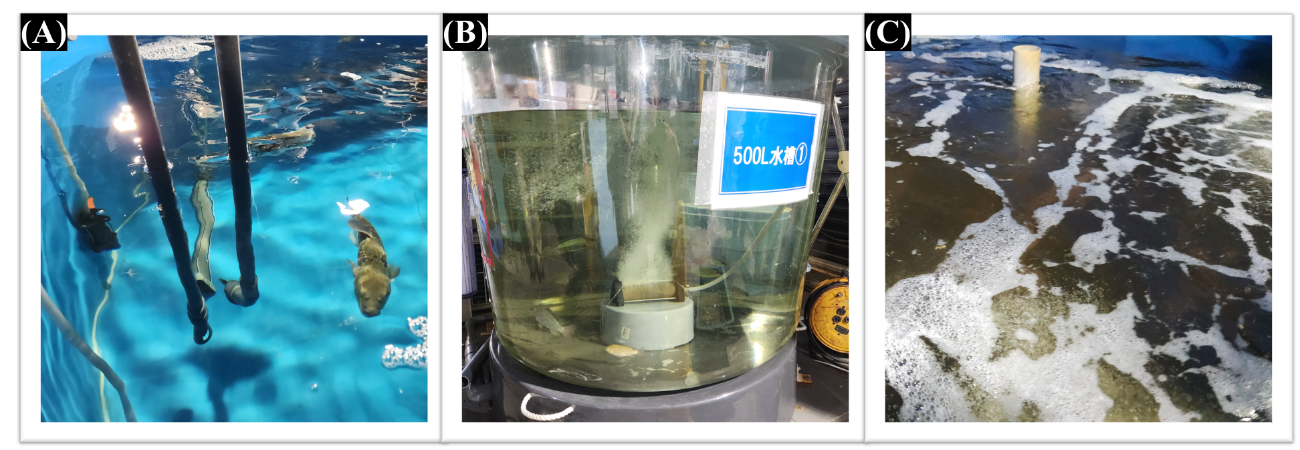
 Supplementary Fig. S1** The aquaculture ponds from where the solid wastes were collected, namely Puffer (A) and Tilapia (B). The wastes are transferred/drained to the waste deposition and collection tank (C).

**Online Resource Supplementary
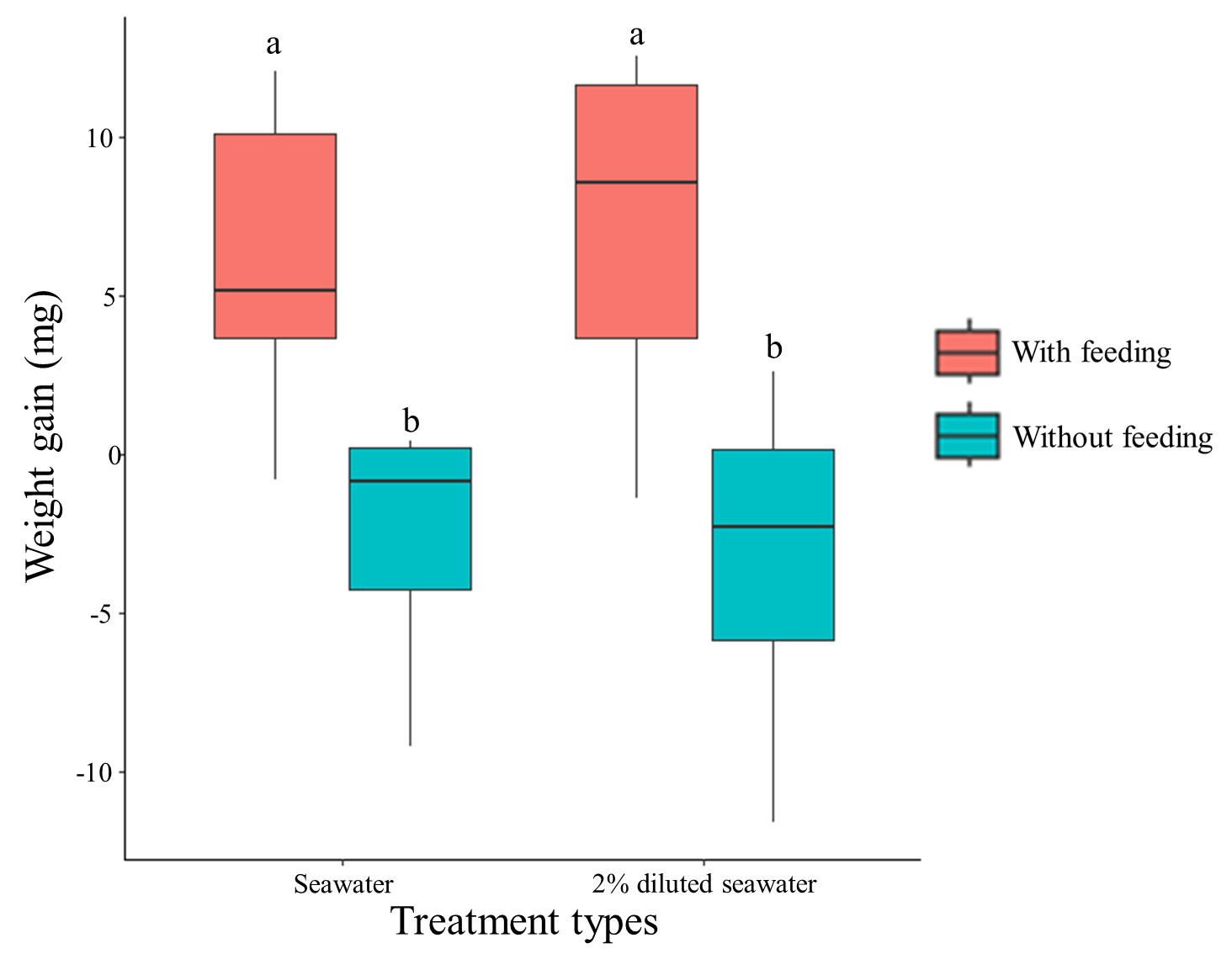
 Fig. S2** Growth pattern of *N. acuminata* juveniles across feeding treatments (e.g., with or without sludge) and standard and 2% diluted seawater (20 g/L; SW) for a week. The alphabetical letters denote the results of multiple comparisons based on generalized linear models (GLMs); those with different letters were statistically distinguishable (GLM: *p*<0.05)*.*

**Online Resource
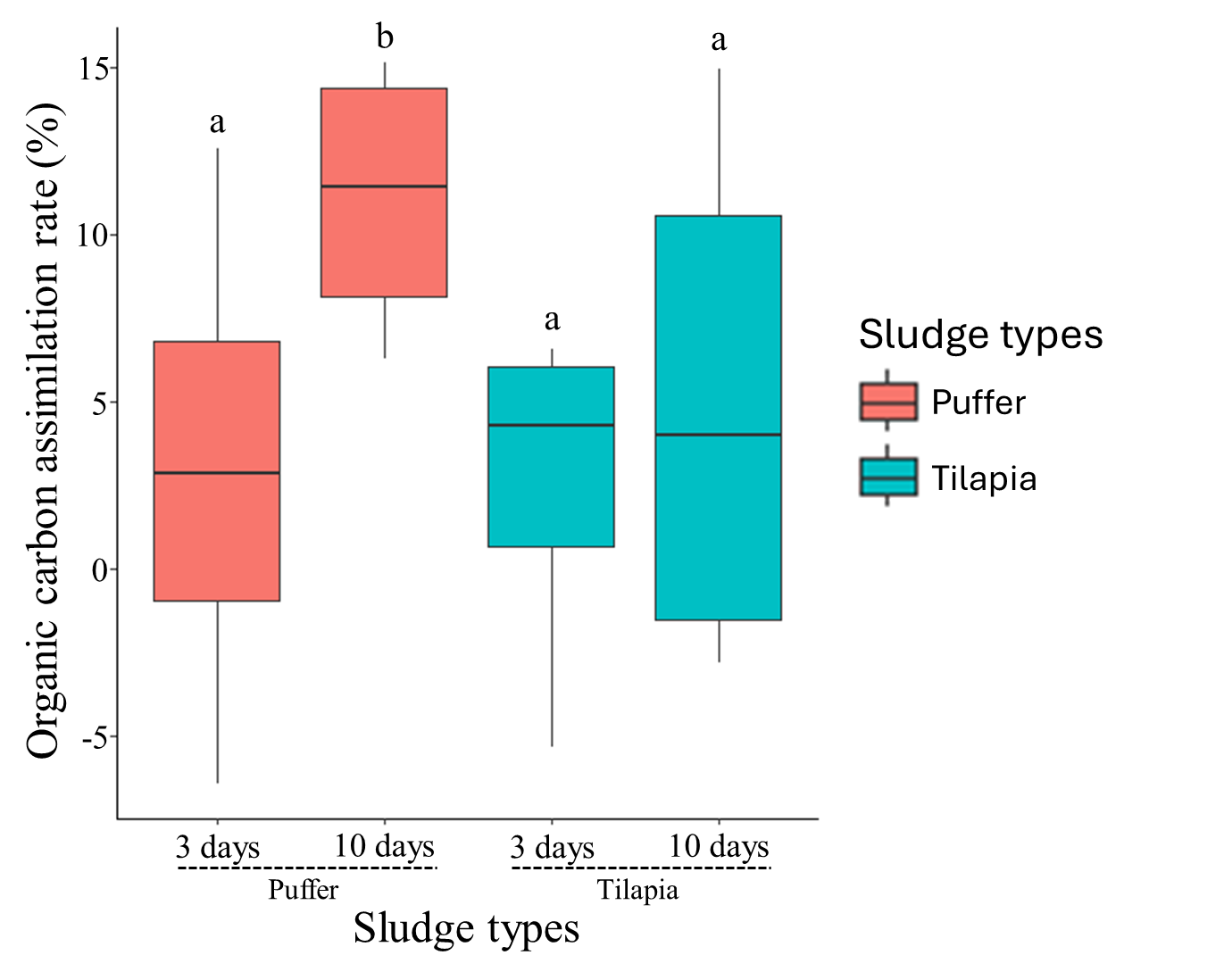
 Supplementary Fig. S3** Carbon assimilation rate of *N. acuminata*. Carbon assimilation rate within the body during the different sludge exposure periods among different sludges and exposure periods. The alphabetical letters denote the results of multiple comparisons based on generalized linear models (GLMs). Those with different letters were statistically distinguishable (*p*<0.05). No statistically significant differences were observed among sludge types; therefore, they are not shown in the figure.

**Online Resource Supplementary Table S1** Table shows the survival percentage across different salinity dilution levels at various observation hours.

| Observation time (hours) | Survival across salinity levels (% dilution) | | | | | | |
| --- | --- | --- | --- | --- | --- | --- | --- |
|  | 0^(5)^ | 0.5^(5)^ | 1^(5)^ | 1.5^(5)^ | 2^(5)^ | 2.5^(5)^ | 3^(5)^ |
| 0 | 100 | 100 | 100 | 100 | 100 | 100 | 100 |
| 3 | 0 | 0 | 100 | 100 | 100 | 100 | 100 |
| 24 | 0 | 0 | 80 | 100 | 100 | 100 | 100 |
| 48 | 0 | 0 | 0 | 100 | 100 | 100 | 100 |
| 72 | 0 | 0 | 0 | 0 | 100 | 100 | 100 |

* Bracketed superscripts denote the number of replicates

**Online Resource Supplementary Table S2** Results of generalized linear mixed models (GLMMs) testing the variabilities in growth (a) and specific growth rate (b) of *N. acuminata* among the treatments (i.e., negative control, puffer, and tilapia sludge), observation weeks, and their interactions. Additional results of GLMMs summarizing the variabilities in growth (c) and specific growth rate (d) of *N. acuminata* among the treatments (i.e., puffer and tilapia sludge), observation weeks, and their interactions. Full models and reduced models were each compared using log-likelihood ratio tests. When full models were insignificant, the first reduced models were compared sequentially with the second reduced models. Superscripts on *p*-values indicate the variables removed from the model when testing those from reduced models by one level. Bold letters for *p*-value denote statistical significance.

| **(a) Explanatory variable** | | |  | | | **logLik** | | **AIC** | | | | ***p*-value** |
| --- | --- | --- | --- | --- | --- | --- | --- | --- | --- | --- | --- | --- |
| *Full model* | | | | | |  | |  | | | |  |
|  | Treatments (T), Weeks (W), T×W | | | | | -831.61 | | 1715.2 | | | | **<0.01** |
| *1^st^ Reduced model* | | | | | |  | |  | | | |  |
|  | T, W | | | | | -846.25 | | 1716.5 | | | |  |
| **(b) Explanatory variable** | | |  | | **logLik** | | | | **AIC** | | ***p*-value** | |
| *Full model* | | | | |  | | | |  | |  | |
|  | Treatments (T), Weeks (W), T×W | | | | -508.89 | | | | 1069.8 | | **<0.05** | |
| *1^st^ Reduced model* | | | | |  | | | |  | |  | |
|  | T, W | | | | -521.40 | | | | 1066.8 | |  | |
| **(c) Explanatory variable** | |  | | **logLik** | | | **AIC** | | | ***p*-value** | | |
| *Full model* | | | |  | | |  | | |  | | |
|  | Treatments (T), Weeks (W), T×W | | | -600.38 | | | 1236.8 | | | 0.55 | | |
| *1^st^ Reduced model* | | | |  | | |  | | |  | | |
|  | T, W | | | -603.31 | | | 1228.6 | | | **<0.01^T^**, **0.001^W^** | | |
| *2^nd^ Reduced model* | | | |  | | |  | | |  | | |
|  | T | | | -623.26 | | | 1254.5 | | |  | | |
|  | W | | | -607.84 | | | 1235.7 | | |  | | |
| **(d) Explanatory variable** | |  | | **logLik** | | | **AIC** | | | ***p*-value** | | |
| *Full model* | | | |  | | |  | | |  | | |
|  | Treatments (T), Weeks (W), T×W | | | -303.59 | | | 627.2 | | | 0.55 | | |
| *1^st^ Reduced model* | | | |  | | |  | | |  | | |
|  | T, W | | | -305.21 | | | 624.4 | | | **<0.05^T^**, **0.01^W^** | | |
| *2^nd^ Reduced model* | | | |  | | |  | | |  | | |
|  | T | | | -311.79 | | | 631.6 | | |  | | |
|  | W | | | -307.17 | | | 626.3 | | |  | | |

| Week | Control/no feeding | Puffer sludge | Tilapia sludge |
| --- | --- | --- | --- |
|  | SGR (% per day) | SGR (% per day) | SGR (% per day) |
| 1^st^ | -2.75 ± 5.12^(10)^ | 6.69 ± 4.29^(10)^ | 7.54 ± 6.71^(10)^ |
| 2^nd^ | -1.05 ± 2.99^(10)^ | 6.38 ± 3.56^(9)^ | 6.79 ± 4.45^(10)^ |
| 3^rd^ | -2.14 ± 3.03^(10)^ | 4.19 ± 2.49^(9)^ | 5.90 ± 2.16^(10)^ |
| 4^th^ | -1.98 ± 2.18^(9)^ | 3.70 ± 1.86^(9)^ | 4.30 ± 1.03^(10)^ |
| 5^th^ | -1.81 ± 1.83^(8)^ | 3.10 ± 1.66^(9)^ | 4.03 ± 1.25^(9)^ |
| 6^th^ | -0.72 ± 1.40^(7)^ | 2.46 ± 1.50^(9)^ | 3.85 ± 1.10^(9)^ |
| 7^th^ | -1.16 ± 1.19^(6)^ | 2.37 ± 1.36^(8)^ | 3.52 ± 0.90^(9)^ |
| 8^th^ | -1.13 ± 1.09^(6)^ | 2.21 ± 1.32^(8)^ | 3.02 ± 0.64^(8)^ |

**Online Resource Supplementary Table S3** A table showing the specific growth rate (i.e., SGR; mean ± standard deviations) of *N. acuminata* juveniles across 8 weeks of experimental period. Negative control denotes the replicates without feeding and sludge.

*Bracketed superscripts denote the number of replicates

**Online Resource Supplementary Table S4** Results of generalized linear models (GLMs) testing the variability between exposed and initial *N. acuminata* juveniles between sludge types (Treatments: Puffer and tilapia sludge types) in terms of stable nitrogen isotope (δ^15^N) (a) and stable carbon isotope ratios (δ^13^C) (b). Full models and null models were each compared using log-likelihood tests. Bold letters for *p*-value denote statistical significance.

| 1. **Explanatory variable** | | | |  | **LogLik** | | | | **AIC** | | | ***p*-value** | |
| --- | --- | --- | --- | --- | --- | --- | --- | --- | --- | --- | --- | --- | --- |
| *Full model* | | | |  | | | |  | | | | |  |
|  | Treatments | | | -43.071 | | | | 102.14 | | | | | **<0.001** |
| *Null model* | | | |  | | | |  | | | | |  |
|  | N.A. | | | -105.892 | | | | 215.78 | | | | |  |
| 1. **Explanatory variable** | |  | **LogLik** | | | | **AIC** | | | ***p*-value** | | | |
| *Full model* | |  | | | |  | | | | |  | | |
|  | Treatments | 4.276 | | | | 7.4486 | | | | | **<0.001** | | |
| *Null model* | |  | | | |  | | | | |  | | |
|  | N.A. | -83.196 | | | | 170.39 | | | | |  | | |

**Online Resource Supplementary Table S5**: Results of generalized linear models (GLMs) testing the differences of isotopic signature in *N. acuminata* juveniles due to the exposure to the puffer sludge in terms of stable nitrogen isotope ratios (δ^15^N) (a) and stable carbon isotope ratios (δ^13^C) (b). Full models and null models were each compared using log-likelihood tests. Bold letters for *p*-value denote statistical significance.

| 1. **Explanatory variable** | | |  | | **LogLik** | | | | **AIC** | | ***p*-value** | | |  |
| --- | --- | --- | --- | --- | --- | --- | --- | --- | --- | --- | --- | --- | --- | --- |
| *Full model* | | |  | | | | |  | | | |  | |  |
|  | | Treatments | -10.09 | | | | | 28.18 | | | | **<0.001** | |  |
| *Null model* | | |  | | | | |  | | | |  | |  |
|  | | N.A. | -19.74 | | | | | 43.48 | | | |  | |  |
| 1. **Explanatory variable** | | | |  | | **LogLik** | | | **AIC** | | | | ***p*-value** | |
| *Full model* | | | |  | | |  | | |  | | | |  |
|  | Treatments | | | 0.19 | | | 7.61 | | | 0.30 | | | |  |
| *Null model* | | | |  | | |  | | |  | | | |  |
|  | N.A. | | | -0.99 | | | 5.99 | | |  | | | |  |

**Online Resource Supplementary Table S6** Results of generalized linear models (GLMs) testing the differences of isotopic signature in *N. acuminata* juveniles due to the exposure to the tilapia sludge in terms of stable nitrogen isotope ratios (δ^15^N) (a) and stable carbon isotope ratios (δ^13^C) (b). Full models and null models were each compared using log-likelihood tests. Bold letters for *p*-value denote statistical significance.

| 1. **Explanatory variable** | | | |  | | **LogLik** | | | **AIC** | | | ***p*-value** |  |
| --- | --- | --- | --- | --- | --- | --- | --- | --- | --- | --- | --- | --- | --- |
| *Full model* | | | |  | | | |  | | |  | |  |
|  | | Treatments | | -15.11 | | | | 38.23 | | | 0.11 | |  |
| *Null model* | | | |  | | | |  | | |  | |  |
|  | | N.A. | | -17.32 | | | | 38.64 | | |  | |  |
| 1. **Explanatory variable** | | |  | | **LogLik** | | | | **AIC** | ***p*-value** | | | |
| *Full model* | | |  | | | |  | | | | |  |  |
|  | Treatments | | -3.05 | | | | 14.11 | | | | | 0.59 |  |
| *Null model* | | |  | | | |  | | | | |  | |
|  | N.A. | | -3.57 | | | | 11.14 | | | | |  |  |

**Online Resource Supplementary Table S7** Results of generalized linear models (GLMs) testing the differences in carbon to nitrogen ratio (i.e., C:N) of *N. acuminata* juveniles in puffer (a) and tilapia (b) sludge. Each model compared samples, including C:N ratios of initial and sludge-exposed *N. acuminata* juveniles, initial sludge, and faecal materials after 10 days of exposure. Full models and null models were each compared using log-likelihood tests. Bold letters for *p*-value denote statistical significance.

| 1. **Explanatory variable** | |  | **LogLik** | | **AIC** | ***p*-value** | |
| --- | --- | --- | --- | --- | --- | --- | --- |
| *Full model* | |  | |  | | |  |
|  | Sample types | 9.24 | | -6.47 | | | **<0.001** |
| *Null model* | |  | |  | | |  |
|  | N.A. | -42.95 | | 89.90 | | |  |
| 1. **Explanatory variable** | |  | **LogLik** | | **AIC** | ***p*-value** | |
| *Full model* | |  | |  | | |  |
|  | Sample types | 6.64 | | -1.29 | | | **<0.001** |
| *Null model* | |  | |  | | |  |
|  | N.A. | -38.54 | | 81.09 | | |  |

**Online Resource Supplementary Table S8** Nitrogen and carbon contents in different samples analysed before and after the sludge exposure experiment (mean ± standard deviation).

| Sludge type | Sample type | Exposure days | N (mg) | C (mg) |
| --- | --- | --- | --- | --- |
| N.A. | *N. acuminata* | 0-day | 0.1 ± 0.01 ^(4)^ | 0.2 ± 0.04 ^(4)^ |
| Puffer | *N. acuminata* | 3-day | 0.1 ± 0.01 ^(4)^ | 0.3 ± 0.03 ^(4)^ |
|  | *N. acuminata* | 10-day | 0.1 ± 0.02 ^(4)^ | 0.3 ± 0.06 ^(4)^ |
|  | Sludge | 0-day | 0.2 ± 0.01 ^(4)^ | 1.2 ± 0.10 ^(4)^ |
|  | Fecal materials | 10-day | 0.1 ± 0.07 ^(4)^ | 0.9 ± 0.56 ^(4)^ |
| Tilapia | *N. acuminata* | 3-day | 0.1 ± 0.02 ^(4)^ | 0.3 ± 0.08 ^(4)^ |
|  | *N. acuminata* | 10-day | 0.1 ± 0.03 ^(4)^ | 0.2 ± 0.1 ^(4)^ |
|  | Sludge | 0-day | 0.3 ± 0.09 ^(4)^ | 2.0 ± 0.59 ^(4)^ |
|  | Fecal materials | 10-day | 0.2 ± 0.12 ^(4)^ | 1.1 ± 0.88 ^(4)^ |

*N.A. denotes initial samples that were not exposed to sludge

*****Bracketed superscripts denote the number of replicates

**Online Resource Supplementary Table S9** Results of generalized linear models (GLMs) testing the differences of nitrogen uptake (a), carbon uptake (b), and organic carbon assimilation (c) by *N. acuminata* between puffer and tilapia fish farm sludge exposure conditions. Full models and null models were each compared using log-likelihood tests. Bold letters for *p*-value denote statistical significance.

| 1. **Explanatory variable** | | | |  | | **LogLik** | | | **AIC** | | | ***p*-value** | | | |  |
| --- | --- | --- | --- | --- | --- | --- | --- | --- | --- | --- | --- | --- | --- | --- | --- | --- |
| *Full model* | | | |  | | |  | | | |  | | |  |  |  |
|  | | Sludge types | | -56.69 | | | 119.39 | | | | **<0.001** | | |  |  |  |
| *Null model* | | | |  | | |  | | | |  | | |  |  |  |
|  | | N.A. | | -62.99 | | | 129.97 | | | |  | | |  |  |  |
| 1. **Explanatory variable** | | |  | | **LogLik** | | | | **AIC** | | | | ***p*-value** | | | |
| *Full model* | | |  | | | | |  | |  | | | |  |  |  |
|  | Sludge types | | -44.87 | | | | | 95.74 | | **<0.001** | | | |  |  |  |
| *Null model* | | |  | | | | |  | |  | | | |  |  |  |
|  | N.A. | | -50.57 | | | | | 105.13 | |  | | | |  |  |  |
| 1. **Explanatory variable** | | |  | | **LogLik** | | | | **AIC** | | | | **p-value** | |  |  |
| *Full model* | | |  | | | | |  | |  | | | |  |  |  |
|  | Sludge types | | -52.83 | | | | | 111.65 | | 0.33 | | | |  |  |  |
| *Null model* | | |  | | | | |  | |  | | | |  |  |  |
|  | N.A. | | -53.31 | | | | | 110.62 | |  | | | |  |  |  |
